# Evaluation of Total Antioxidant Capacity of Saliva in High School Students

**DOI:** 10.5539/gjhs.v8n4p89

**Published:** 2015-07-30

**Authors:** Mahmoud Rahmani, Vahideh Ghoorchi, Fatemeh Rezaei, Asad Vaisi Raygani

**Affiliations:** 1 Oral Medicine Department, School of Dentistry, Kermanshah University of Medical Sciences, Kermanshah, Iran; 2 School of Dentistry, Kermanshah University of Medical Sciences, Kermanshah, Iran; 3 Biochemistry Department, School of Medicine, Kermanshah University of Medical Sciences, Kermanshah, Iran

**Keywords:** Dental caries, total antioxidant capacity, oxidative stress

## Abstract

**Background and Objective:**

Imbalance between oxidative stress and saliva antioxidants plays a major role in initiation and spread of dental caries. The aim of this study was to evaluate the total antioxidant capacity (TAC) of saliva in dental caries.

**Methods:**

In this case-control study which employed high school students (14-18 years), the un-stimulated saliva samples of 60 students without dental caries (control group) and 60 students with dental caries (with at least 5 teeth levels of dental caries) were gathered. Each group comprised of males (half of cases) and females (half of cases). TAC of saliva was measured by Zellbio® (Netherlands) in terms of micmol/L. The data were analyzed using the SPSS software (ver. 17.0) and t-test with considering significance level at 0.05.

**Results:**

TAC of saliva was significantly lower in cases with dental caries (0.256±0.106) compared to those without dental caries (0.396±0.077); P< 0.001. There was no statistically significant difference of TAC of saliva between males (0.319±0.119) and females (0.333±0.113) irrespective of dental caries (P=0.507).

**Conclusion:**

The results of the study indicated that there was a reverse association between dental caries and TAC of saliva.

## 1. Introduction

Dental caries is one the most common oral health disease and it's prevention is the important strategies in many countries (Hegde et al., 2009).

Saliva is a complex fluid in the oral cavity, products by major and minor salivary glands and has the important role in oral health and specially prevention of dental caries (Lima et al., 2010; Mandel, 1993; Animireddy et al., 2014).

Saliva composition, naturally or under certain conditions, varies in different individuals (Reyes et al., 2014). Total antioxidant capacity (TAC) is the total material in bodily fluids which possess antioxidant properties (Shafer et al., 2009).

Saliva contains antioxidant similar to other bodily fluids. TAC of saliva is comprised of enzyme elements such as superoxide dismutase, peroxidase, and non-enzyme elements including uric acid, vitamin C, reduced glutathione, and oxidized glutathione (Bakhtiari et al., 2012; Pendyala et al., 2013).

There are many studies about role of saliva antioxidant and some of them claimed recently that imbalance between free radicals and antioxidants in saliva may play significant roles in beginning and developing of dental caries (Kamodyová et al, 2013, Gopinath et al., 2006; Battino et al., 2002).

Thus we aimed to find whether there is a relationship between TAC of whole saliva and dental caries in males and females students.

## 2. Materials and Methods

In this case-control study, the observation was done among male and female high school students (14-18 years) of Kermanshah city, Iran. The sample size was obtained using the following formula:


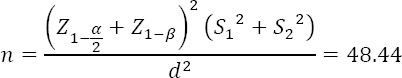


Considering 95% confidence level, power of 90%, and accuracy of 0.3, and standard deviation of dental caries in females (0.048) and males (0.043) (Dodwad et al., 2011), the sample size was calculated to be at least 49 cases in each group. We recruited 60 subjects, by using cluster sampling method to participating students from 1-4 levels of high school randomly.

Study Inclusion Criteria:

1) Being generally healthy
2) No history of having periodontal disease
3) Having at least 5 decayed tooth surfaces (case group)
4) Having no dental caries DMFT=0 (control group)

Study Exclusion Criteria:

1) Having a systemic illness
2) Having pathologic lesion in oral cavity
3) Periodontal disease
4) Any drug use (within last three months)
5) Smoking
6) Bad oral hygiene

The study protocol was approved by the Ethics Committee of Kermanshah University of Medical Science, Kermanshah, Iran (p/7/420/40325). Written informed consent was obtained from the students. All students were examined by single examiner (general dentist). The examination was performed by mirror, explorer and flash light. Caries-active students had at least five decayed tooth surfaces (Ahmadi-Motamayel et al., 2013;Tulunoglu et al., 2006). Caries-free group were students that did not have any caries, filling or extracted tooth, (DMFT = 0). Students with dental caries and without caries (case and control groups) were matched by sex, age, municipal regions of high school, brushing frequency (twice a day).

120 students in 4 groups (30 in each group) were examined:

Group 1: 30 male students with at least 5 decayed tooth surfaces;

Group 2: 30 male students without dental caries;

Group 3: 30 female students with at least 5 decayed tooth surfaces;

Group 4: 30 female students without dental caries.

Obtaining saliva samples: Un-stimulated saliva samples were collected in the morning. The students were asked to brush their teeth and avoid eating or drinking for at least 90 minutes before saliva sampling. The saliva sampling was done while the patient was seated and with bending head forward. In this state, the student emptied his/her saliva in a glass container. The samples were immediately placed at packets containing ice and were transferred to the laboratory (Navazesh, 1993).

All samples were centrifuged (200 rpm) for 10 minutes to separate all debris. Then, the sample was stored at – 20°C until being examined in the laboratory.

The TAC was measured using Zellbio® kits (Netherlands) defining the levels in the unit of micmol/L.

The Principles and Method of Measuring TAC

To measure the TAC, the TAC kit (Zillbio®, Netherlands) was used. The measurement was done using the FRAP method. In this method, in the presence of TPTZ (2, 4, 6-tvi-pyvidyl-s-wiazin), the complex of Fe^+3^-tptz in the presence of anti-oxidants is converted to Fe^+2^-tptz. This complex has a pinkish color and its color intensity has direct relationship with TAC level. This color has the maximum absorption at 520 nm wavelength (Benzie et al., 1996).

### 2.1 Statistical Analyses

The data were analyzed using the SPSS Software (ver. 17.0) applying the Students t-test. The significance level was set at 0.05.

## 3. Results

**Figure 1:**
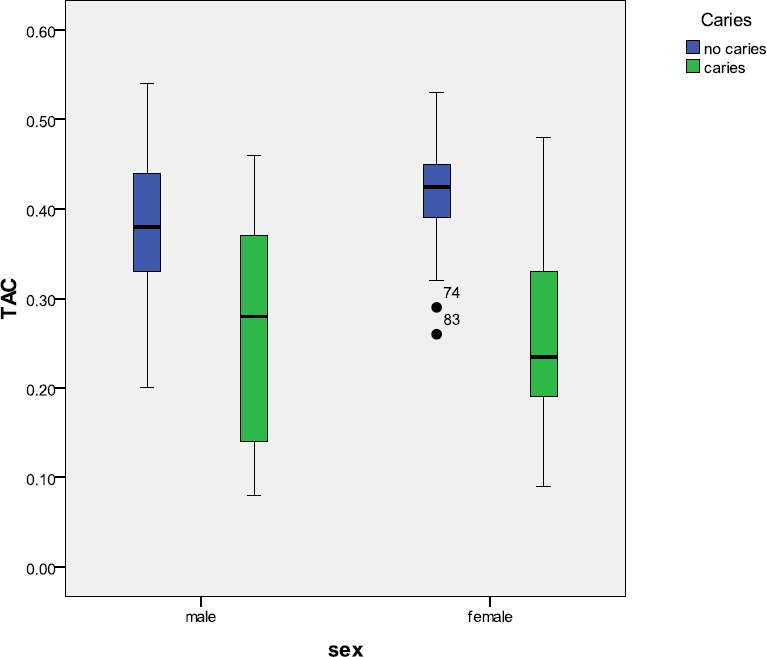
Boxplot diagram showing total antioxidant capacity of saliva (micmol/L) in students with and without dental caries

In Figure 1, the TAC (micmol/L) of the saliva of male and female students based on the presence of dental caries is presented.

According to Table 1, TAC of saliva in those without dental caries was significantly higher (0.396±0.077) compared to those with dental caries (0.256±0.106); P<0.001.

**Table 1:** Comparison of total antioxidant capacity of saliva (micmol/L) between students with and without dental caries

	Without dental caries	With dental caries	P value*
Male	0.377 (±0.086)	0.260 (±0.120)	< 0.001
Female	0.414 (±0.063)	0.252 (±0.092)	< 0.001
Total	0.396 (±0.077)	0.256 (±0.106)	< 0.001

* t-test.

In Table 2, the saliva TAC is compared between male and female students. TAC level in male students was 0.319±0.119 and in female students it was 0.333±0.113 without any significant difference (P= 0.507).

**Table 2:** Comparison of total antioxidant capacity of saliva (micmol/L) between male and female students

	Without dental caries	With dental caries	Total
Male	0.377 (±0.086)	0.260 (±0.120)	0.319 (±0.119)
Female	0.414 (±0.063)	0.252 (±0.092)	0.333 (±0.113)
P value*	0.064	0.756	0.507

* t-test.

## 4. Discussion

In the current study, the TAC was measured in un-stimulated sample of saliva. Moore (1994) and Krawczyk (2014) by studying TAC in un-stimulated and stimulated saliva samples reported that TAC was higher in un-stimulated saliva and therefore it appears that un-stimulated saliva is more accurate to evaluate the anti-oxidant properties.

The current findings showed that TAC of saliva in those with dental caries was significantly lower compared to those without dental caries. Similar to our findings, Krawczyk (2014) reported that in patients (15-17 years) with increasing the number of caries, stimulated and un-stimulated saliva anti-oxidant level significantly decreased. In another study, Krawczyk (2012) showed decreased TAC of saliva in subjects with dental caries. The reduction of salivary TAC in students with dental caries may be related to enhanced activity of neutrophils and monocytes in the oral cavity which produce reactive oxygen species (ROS) in the presence of bacteria. In other words, enhanced production of ROS leads to decreased TAC of saliva (Krawczyk et al., 2014).

The anti-oxidants present in the saliva are the first defense mechanism against oxidative stress due to free radicals (Hegde et al., 2012; Shetty et al., 2011).

There are many studies that reported decreased anti-oxidants capacity of saliva has important role in initiation and spread of many diseases of oral cavity including oral lichen planus (Shirzad et al., 2014), periodontal disease (Sculley et al., 2003; Tóthová et al., 2013; Azizi A et al., 2014), due to oxidative stress. So we propose that like these conditions reduction in TAC of saliva (as a protective system) may have a significant role in dental caries because of the destructive factors include free radicals or non-radical oxygen derivatives.

In contrast to the current findings, Dodwad (2011) reported higher TAC in children and teenagers (age range of 7-10 years and 11-14 years) with dental caries in comparison to those without dental caries. Also, in Kumar (2011) study, it was reported that in children aged 3-5 years, severe early childhood caries (S-ECC) was associated with higher TAC level of saliva and a direct linear relationship existed between S-ECC severity and TAC level. The relationship between TAC with dental caries has been reported in adults as well. Hegde (2013) by studying adult cases found that a linear relationship existed between TAC of saliva and dental caries and with worse dental caries, TAC increased.Some mechanisms have been proposed in explaining why TAC could be higher in patients with dental caries. For example, Mahjoub (2014) proposed that higher TAC level in children with S-ECC is a compensatory mechanism against oxidative stress. Moore (1994) attributed higher TAC level of saliva in patients with dental caries to their diet. These experts stated that saliva TAC is a combination of endogenous and food-derived anti-oxidants. Uric acid, as the major anti-oxidant of the saliva composes more than 85% of saliva TAC is derived from foods and mainly from sugars. Therefore, consumption of sugars not only enhances the risk of dental caries, but also contributed to higher TAC level of saliva.

The controversy seen in different studies can be attributed to different methods of measuring TAC, age discrepancy, the severity of dental caries, and tooth stage. According to study of Uberos (2008), tooth stage is an important factor that affects the relationship between TAC and dental caries. They observed that TAC of saliva in patients with deciduous teeth caries was significantly higher than those without dental caries. But in permanent teeth, no significant relationship was detected between dental caries and TAC of saliva.

According to the presented findings, like the study of Tulunoglu (2006) decreased TAC of saliva related to dental caries was not affected by gender of the students.

But in contrary to what we observed, Ahmadi-Motamayel (2013) reported that TAC of saliva in males with dental caries was higher in comparison to females with dental caries. They suggested, hormonal changes, and possibly diets may change the TAC of saliva.

**Limitation and Suggestion**

As a limitation, the severity of dental caries, flow rate, PH of saliva and antioxidants of serum was not determined. We recommend that these factors be accounted in future studies.

There is a direct relationship between saliva and serum antioxidants, (Lumikari et al., 2000; Reznick et al., 2006; Malekirad et al., 2005). So further studies can be conducted on the protective effect of antioxidant –containing medication to develop new approach to prevent dental caries.

## 5. Conclusion

The current study showed that TAC of un-stimulated saliva in students with dental caries was significantly lower compared to the subjects without dental caries. The decreased saliva TAC was not influenced by gender.
